# Steroid-Refractory Gut Graft-Versus-Host Disease: What We Have Learned From Basic Immunology and Experimental Mouse Model

**DOI:** 10.3389/fimmu.2022.844271

**Published:** 2022-02-18

**Authors:** Qingxiao Song, Ubaydah Nasri, Defu Zeng

**Affiliations:** ^1^ Arthur D. Riggs Diabetes and Metabolism Research Institute, The Beckman Research Institute, City of Hope National Medical Center, Duarte, CA, United States; ^2^ Hematologic Malignancies and Stem Cell Transplantation Institute, City of Hope National Medical Center, Duarte, CA, United States; ^3^ Fujian Medical University Center of Translational Hematology, Fujian Institute of Hematology, and Fujian Medical University Union Hospital, Fuzhou, China

**Keywords:** hematopoietic cell transplantation, steroid-refractory graft versus host disease, experimental mouse model, T cells, IL-22

## Abstract

Intestinal graft-versus-host disease (Gut-GVHD) is one of the major causes of mortality after allogeneic hematopoietic stem cell transplantation (allo-HSCT). While systemic glucocorticoids (GCs) comprise the first-line treatment option, the response rate for GCs varies from 30% to 50%. The prognosis for patients with steroid-refractory acute Gut-GVHD (SR-Gut-aGVHD) remains dismal. The mechanisms underlying steroid resistance are unclear, and apart from ruxolitinib, there are no approved treatments for SR-Gut-aGVHD. In this review, we provide an overview of the current biological understanding of experimental SR-Gut-aGVHD pathogenesis, the advanced technology that can be applied to the human SR-Gut-aGVHD studies, and the potential novel therapeutic options for patients with SR-Gut-aGVHD.

## Introduction

Allogeneic hematopoietic stem cell transplantation (allo-HSCT) is a curative therapy for hematological malignancies, but graft-versus-host disease (GVHD) remains the major obstacle for the widespread application of allo-HSCT (1–7). Systemic glucocorticoid (GC)-based immunosuppressant therapies were applied more than a decade ago and are now a primary therapy for acute GVHD (aGVHD) management (8–14), which is effective in approximately 50% of cases, with a robust response being observed in approximately one-third of patients (15, 16). The gastrointestinal (GI) tract is a prominent target of aGVHD, and it can be as high as 60%, although the reported frequencies of Gut-aGVHD varied (17–20). Due to this, the severity of Gut-aGVHD determines the outcome of allo-HSCT (20). Approximately 15% of aGVHD patients developed into steroid-resistant or steroid-refractory Gut-aGVHD (SR-Gut-aGVHD) (17, 21–23), which is defined as no improvement of clinical symptoms after 4 weeks of high-dose GC treatment, most often with prednisone at 1.0–2.0 mg/kg per day (8, 23, 24). The prognosis of SR-Gut-aGVHD patients is poor, especially in cases of grade III to IV Gut-GVHD, with more than 75% mortality (8, 25). Despite considerable progress and expansion of the therapeutic armamentarium, including antibodies against CD3/CD7, α4β7 integrin, and CD30, as well as targeting the IL-6/IL-6R signaling pathway, the lack of effective treatment remains a major issue (16, 26–30). More recently, a single-arm phase II study (REACH-1) showed a promising effect of JAK1/2 inhibitor, ruxolitinib, for the treatment of SR-aGVHD (31). So far, ruxolitinib is the only Food and Drug Administration (FDA)-approved agent for the second-line treatment of SR-GVHD.

To timely capture the therapeutic opportunity for the benefit of Gut-aGVHD patients, the identification of predictive biomarkers for Gut-aGVHD development and/or prognosis has been a central issue in the field. For example, markers that are specific for Gut-GVHD including regenerating islet-derived 3-α (REG3α) and TIM3 have been identified (25, 32, 33). In addition, clinical trials with the Mount Sinai Acute GVHD International Consortium (MAGIC) algorithm have identified patients at high risk for developing SR-Gut-GVHD as early as 7 days after hematopoietic cell transplantation (HCT), based on the extent of intestinal crypt damage as measured by the concentrations of 2 serum biomarkers, ST2 and REG3α (34–37). Another study that examined markers of endothelial toxicity found follistatin and endoglin to be associated with higher levels of grade III to IV aGVHD and non-relapsed mortality (NRM) (38). Based on these discoveries, a trial of a-1-antitrypsin (AAT), a serine protease inhibitor with demonstrated activity against GVHD, was conducted in patients at high risk for developing SR-GVHD. The trial concluded that real-time biomarker-based risk assignment is feasible early after allogeneic HCT, but the dose and schedule of AAT did not change the incidence of SR-aGVHD (39). Overall, in the absence of a clearly defined resistance mechanism, alternative therapeutic options remain an open-ended question (8).

Experimental models have largely contributed to our understanding of aGVHD pathophysiology (24, 40, 41), while information about the disease’s mechanisms has been gathered from human studies as well (42). Over the past years, many attempts were made to identify the factors associated with poor survival and low response rates in patients with SR-Gut-aGVHD (21, 43). However, due to the lack of an adequate murine model, the pathophysiology of SR-Gut-aGVHD remains largely unknown. Instead of extensively reviewing previous literature, we intend to highlight new insights into SR-Gut-aGVHD pathogenesis provided by murine models and factors, which are likely involved in the pathogenesis of SR-Gut-aGVHD, including cytokines produced by alloreactive T cells in response to GCs, the influence of microbiome and metabolites in Gut-GVHD, and the suppressive role of myeloid-derived suppressor cells (MDSCs). In the end, we will propose potential therapeutic targets for clinical treatment based on what we have learned from basic immunology and any available experimental data.

## New Insights Into Steroid Refractory-Gut-Acute Graft-Versus-Host Disease Provided by Newly Established Murine Models

Medical scientists have been attempting to understand the pathogenesis of SR-Gut-aGVHD for decades, and the progress is gradual. Despite the extensive development of murine models for aGVHD (44), a lack of clinically relevant animal models limits our understanding of the pathophysiology of SR-GVHD. Several attempts have been made to establish an experimental mouse model for SR-GVHD research (45, 46). One of the studies aimed at establishing SR-GVHD murine models using two different clinically relevant models—MHC matched multiple minor antigens (miHAs) mismatched model (C3H.SW (H-2^b^) donors to C57BL/6 (H-2^b^) recipients), and MHC mismatched haploidentical model (C57BL/6 (H-2^b^) donors to B6D2F1 (H-2^b/d^) recipients), as well as two different treatment intervention schemes [early and late dexamethasone (DEX) treatments (45)]. There is evidence that animals can experience SR-GVHD regardless of when steroids are administered post-allo-HSCT (45). Compared to steroid-responsive animals, an overall increase in GVHD-specific histopathological damage to target organs was observed in SR-GVHD animals, although the differences between steroid-refractory and steroid-responsive animals in regard to donor T-cell characteristics were not statistically significant, suggesting that donor T cell-independent mechanisms may play an increasingly important role in the pathogenesis of SR-GVHD than was previously suspected (45).

Consistent with this mouse study, tissue transcriptomics analysis from GVHD patients also showed no differences in T cells including CD8^+^ T cells, naïve CD4^+^ T cells, and memory CD4^+^ T cells between the new onset of aGVHD and SR-aGVHD, but multiple T cell-independent mechanisms were found to be related to SR-aGVHD (47). The study found that gut tissue repair-associated gene amphiregulin (AREG) and the aryl hydrocarbon receptor (AhR) expression levels were increased at aGVHD onset and remained elevated in SR-Gut-aGVHD, indicating potential interaction of host mucosa with microbiota (47). The study also identified higher expression levels of metallothioneins, metal-binding enzymes induced in stress responses, and M2 macrophage genes in SR-Gut-aGVHD (47). Furthermore, poorly surviving patients showed an indication of greater DNA damage and a distinct microbial signature at aGVHD onset as compared to prolonged surviving patients (47). However, IL-22-producing T cells were not evaluated in those studies, and although AhR is the critical nuclear factor for regulating IL-22-producing T subsets (48–51), an increase of AhR expression was observed in the SR-Gut-GVHD target tissues of patients (47), and IL-22 from donor T cells were reported to augment Gut-aGVHD in murine model (52, 53).

Meanwhile, we recently developed an MHC-mismatched murine model of SR-Gut-aGVHD (C57BL/6 (H-2^b^) donors to BALB/c (H-2^d^) recipients). Briefly, a single administration of DEX on day 3 post-HSCT was found to ameliorate aGVHD, while multiple DEX administrations on days 3, 10, 15, and 20 were not found to further improve neither GI-GVHD-associated clinical symptoms (e.g., diarrhea and body weight loss) nor overall survival. These mice that received sustained DEX treatment were revealed as experiencing SR-Gut-aGVHD (46). Following the establishment of a highly relevant model, we analyzed the role of different Th/Tc subsets in the mesenteric lymph nodes and colon tissues. We found that prolonged DEX treatment (i.e., those which resulted in SR-Gut-aGVHD) led to an increase of Th/Tc22 cells in the colon as compared to a single DEX prescription. A similar increase in Th/Tc22 cells was also observed in a Xeno-GVHD model where human peripheral blood mononuclear cells (PBMCs) were infused. By neutralizing IL-22 with a monoclonal antibody specific against IL-22 and by using IL-22-deficient T cells, we validated the role of IL-22 in the development of SR-Gut-aGVHD (46). Our study indicated that prolonged DEX treatment preferentially expands IL-22-producing CD4^+^ and CD8^+^ T cell subsets such as Th/Tc22, and overproduction of IL-22 causes dysbiosis. Prolonged DEX treatment also resulted in the loss of CX3CR1^hi^ mononuclear phagocytes (MNPs) and the dysfunction of MNPs. These new observations are in line with previous reports (52–54). These new observations also identified the potential missing role of IL-22-producing T cells in the murine models and patients of SR-Gut-GVHD pathogenesis. We are now testing whether the expansion of IL-22-producing T cells and loss of CX3CR1^hi^ MNP cells observed in the murine models truly reflect SR-Gut-aGVHD pathogenesis in human.

## Distinct Sensitivity of Th Subsets and Cytokine Pathways to Glucocorticoid Suppression

As apoptosis of alloreactive T cells results in GVHD amelioration, early *in vitro* experiments primarily explored GC-induced apoptosis of human T cells by mixed lymphocyte reaction (MLR) and found that T cells undergo GC-induced apoptosis (55). Various evidence suggests that GCs can cause cell death through various pathways, resulting in apoptotic or necrotic morphologies depending on the presence or absence of apoptotic machinery. These apoptotic morphologies might be caused by the regulation of apoptosis genes, such as those from the Bcl-2 family (56). The necrotic morphologies might also be the result of detrimental effects of GCs on cell function, possibly due to the effect of GC receptor autoinduction (57). Furthermore, GC-induced apoptosis is also inhibited by the cytokines IL-2, IL-4, IL-10, and IL-12 (58–60).

Conditioning chemoradiotherapy can induce tissue damage leading to the release of inflammatory cytokines that initiate the first phase of immune response and reinforce Th/Tc lineage differentiations after allo-HSCT (61, 62). Several studies have determined the susceptibility of Th subsets to GC-induced apoptosis. In general, Th1 cells are sensitive to GCs, which not only reduce their cytokine production but also increase in their apoptosis (56, 63). Th2 cells are less sensitive, with a reduction of their cytokine production but little increase in their apoptosis. Th17 cells appear to be resistant to GCs, with little reduction in their cytokine production or their increase in apoptosis (56, 64). It is worth noting that induction of the proapoptotic protein, BIM, by GCs makes Th1 cells more sensitive to GCs, whereas high levels of anti-apoptotic proteins like BCL-2 make Th17 cells more resistant to GCs (56). Apart from the effects of GCs on Th cells, a lack of consensus exists in current studies regarding the effects of GCs on regulatory T cells (Tregs). Several reports have shown that GCs induce apoptosis in Tregs (65–67), whereas others suggest that GCs expand Tregs (68). Both Th1 cells and Tregs are protected from GC-induced apoptosis by IL-2, while Th2 cells are protected by IL-4 (63). Additionally, IL-15 and IL-7 protect both Tregs and effector T cells from GC-induced apoptosis (65).

There are many ways that GCs can influence the apoptosis of different Th subsets as well as influence the many different pathways in which cytokines are produced by those Th subsets. Some *in vitro* studies indicated that GCs decrease IFN-γ production by T cells from both healthy donors and patients with rheumatoid arthritis (69, 70). Similar results were found in the studies of GCs’ impact on Th2 cytokines IL-4, IL-5, and IL-13 (71–73). In primary Th17 cells, IL-17A and IL-17F productions are resistant to GC suppression, but the effect of both cytokines can be reduced by GCs (74–76). Accordingly, the resistance of IL-17A and IL-17F to GCs is highly dependent on the tissue microenvironment of those Th17 cells. A combination of LPS and dopamine resulted in increased IL-6 production, which in turn induced GCs resistance in Th17 cells (77, 78), while IL-6R-blocking antibodies allow GCs to suppress IL-17A (79). Furthermore, IL-22 that is shared by distinct Th subsets, such as Th17, Th22, and Th1 (80), has been reported to respond in a different manner to GCs. In patients with immune thrombocytopenia, for example, Th22 cells and their IL-22 production are decreased by GCs (81). Moreover, it has been demonstrated that GCs are able to suppress IL-22 production in a mouse model of bacterial infection, while they are unable to suppress IL-22 production in a Th17-adoptive transfer model of airway inflammation (82, 83). Consistently, our recent study found that the expansion of IL-22-producing allogeneic T cells was associated with the pathogenesis of SR-Gut-aGVHD in our experimental mouse model, and the elevated IL-22 level led to dysbiosis and further augmented Gut-GVHD (46). These results suggest that IL-22 could be either a friend or a foe depending on the disease context.

In addition, GCs can increase the amount of neutrophils in the blood (84), expand all stages of neutrophil development (85), and inhibit neutrophil apoptosis (86). Neutrophils have been found to facilitate the development of Gut-GVHD (87, 88). Besides amplifying tissue damage *via* reactive oxygen species (ROS) production (87), neutrophils not only promote differentiation and chemotaxis of Th17 cells (89, 90) but also augment T-cell expansion through antigen presentation on major histocompatibility complex (MHC)-II (88). Thus, GCs may trigger a positive feedback loop between pathogenic T cells and neutrophils in the context of SR-Gut-aGVHD.

There appears to be a gradient of GC sensitivity among Th cells and their cytokine pathways. Considering that Th subsets have an extensive network of cross talk, the degree of sensitivity may vary depending on the disease condition and the tissue microenvironment. While cytokines IL-17A, IFN-γ, and IL-4 are generally considered as antagonistic, cross talk between unique Th cells can lead to synergistic responses. It has been reported that GC-resistant asthma patients have elevated levels of both IL-17 and IFN-γ (91). Furthermore, T cells that express both IL-4 and IL-17 were identified in a different group of patients with GC-resistant asthma (92). In the setting of GVHD, Tc17 cells were found to express high levels of multiple prototypic lineage-defining transcription factors (TFs) (e.g., RORγt and T-bet) and cytokines (e.g., IL-17A, IL-22, IFN-γ, granulocyte-macrophage colony-stimulating factor (GM-CSF), and IL-13) (93). A recent study may provide an explanation for this cross talk: Puleston et al. found that CD4^+^ helper T-cell lineage fidelity is governed by polyamine metabolism, and polyamine-deficient T cells showed severe deficiency to adopt correctly selected subsets, resulting in the simultaneous expression of multiple lineage-defining TFs and cytokines (94).

Therefore, it would be worthwhile to determine whether Th cells having dual or multiple identities are sensitive to GCs based on their cell identity and their cytokine profile. In the context of SR-Gut-aGVHD, it will be necessary to see whether long-term treatment of GCs results in a shift from GC-sensitive to GC-resistant Th subsets and whether the GC-resistant Th subsets have irregular lineage phenotype.

## Dual Role of IL-22 in Promoting Tissue Regeneration and Inflammatory Response in the Context of Steroid Refractory-Gut-Acute Graft-Versus-Host Disease

The role of IL-22 in the context of GVHD is controversial. It was reported that IL-22 produced by donor T cells can enhance Gut-aGVHD (52, 53), while IL-22-deficient grafts can increase Tregs in the spleen and mesenteric lymph nodes of recipient mice (52). Donor-derived IL-22 may also have a pathogenic effect *via* its synergistic effects, with type I IFNs released during allogeneic immune response (53). Additionally, our most recent study showed that alloreactive donor T cell-derived IL-22 augmented gut dysbiosis and enhanced bacterial translocation into the liver of mice with SR-Gut-aGVHD. In contrast, infusion of IL-22-deficient donor T cells was able to reverse dysbiosis and prevent bacteria translocation, thus ameliorating SR-Gut-aGVHD (46).

In another study, Hanash et al. found that the IL-22 receptor is expressed on intestinal stem cells (ISCs) as well as their downstream progenitors. Deficiency of recipient-derived IL-22 resulted in increased crypt apoptosis, depletion of ISCs, loss of epithelial integrity, and augmentation of Gut-aGVHD (95). *In vivo* IL-22 administration after allo-HSCT promoted the recovery of ISCs, increased epithelial regeneration, and reduced intestinal pathology and mortality in an MHC-matched mice model (LP (H-2^b^) donors to C57BL/6 (H-2^b^) recipients) (96). By using *ex vivo* organoid culture, they found that group 3 innate lymphoid cells (ILCs) secreted IL-22, which stimulated the growth of small intestinal organoids. Moreover, recombinant IL-22 treatment increased STAT3 expression in Lgr5^+^ ISCs, which is critical for IL-22-mediated epithelial regeneration (96). A phase 2 clinical study involving IL-22 IgG2-Fc (F-652) treatment on subjects with grade II to IV lower Gut-aGVHD was conducted, and the preliminary data showed response to treatment was 7/12 (58%) with high-risk, 3/4 (75%) with intermediate-risk, and 4/4 (100%) with low-risk biomarkers based on Ann Arbor Risk (97).

However, the role of IL-22 has yet to be fully established in human GVHD, although it appeared to be elevated in both aGVHD and chronic GVHD (cGVHD). During aGVHD, Brüggen et al. observed an increase in IL-22 messenger RNA and IL-22-producing CD4^+^ T cells in the skin (98). Similarly, Lounder et al. reported that higher plasma IL-22 level is positively correlated with the incidence of Gut-GVHD in children (99). In cGVHD, on the other hand, Gartlan et al. showed that donor CD4^+^ T cell-derived IL-22 significantly exacerbated cutaneous cGVHD, and high levels of both IL-17A and IL-22 expressions were present in the skin of cGVHD patients (100). Due to the degree of MHC disparity, bone marrow (BM) and T-cell doses, genetic manipulation of specific cell populations, and control of the microbiome environment, paradoxical results were observed regarding the role of IL-22 in experimental GVHD. Given the dual proinflammatory and anti-inflammatory properties of IL-22 in the context of GVHD, it is likely that this duality will be the greatest obstacle to its development as a therapeutic target.

To date, several underlying mechanisms have been identified in the pathogenesis of SR-Gut-aGVHD such as the key role of T-cell responses, which relate to donor T-cell characteristics, inflammatory cytokine levels, and timing of steroid initiation (45, 46). On the other hand, the approaches aiming to improve tissue regeneration are also an important direction for SR-GVHD therapy. In addition to IL-22, interferon-lambda (IFNλ, IL-28/IL-29) has been identified as a key protector of Gut-GVHD immunopathology (101). In this study, PEGylated IL-29 (PEG-rIL-29) treatment was found to improve survival, reduce GVHD severity, and enhance epithelial proliferation and ISC-derived organoid growth after allo-HSCT (101). However, the question of whether IFNλ contributes to the protection against SR-Gut-aGVHD requires further study. Moreover, intestinal goblet cells (102) and glucagonlike-peptide-2 (GLP-2) produced by intestinal L cells (103) were found to play a protective role in the setting of GVHD. Goblet cell damage in the large intestine correlated with poorer survival of patients after allo-HSCT (102). Pretransplant administration of interleukin-25 (IL-25), a growth factor for goblet cells, prevented bacterial translocation, reduced plasma concentrations of interferon-γ (IFN-γ) and IL-6, and ameliorated GVHD in mouse model (102). Reduced intestinal GLP-2 levels were found in both mice and patients with GVHD (103), treatment with GLP-2 agonist, teduglutide, not only reduced aGVHD but also improved the outcome of SR-GVHD without compromising graft-versus-leukemia (GVL) effects in multiple mouse models (103), suggesting that GLP-2 agonist could be a novel immunosuppressive approach in SR-GVHD therapy to be tested in future clinical trials.

Therefore, to identify potential therapeutic targets, further studies are required to examine the complex immune cell interaction networks involving T cells, neutrophils, monocytes, and non-immune cells such as goblet cells, Paneth cells, and ISCs.

## Microbial Community and Their Metabolites in Regulating Steroid Refractory-Gut-Acute Graft-Versus-Host Disease

There is also an emerging understanding regarding the role of the gut microbiome in Gut-GVHD in both murine and human studies (104–106). Loss of flora diversity following allo-HSCT is associated with the development of Gut-GVHD in addition to increased mortality risk (107, 108). Some commensal microbiota was found to expand Tregs (109), which is beneficial to the outcome of Gut-GVHD, while pathogens such as *Enterococcus* were considered as aGVHD risk factor (110).

One of the mechanisms by which the gut microbiome influences Gut-GVHD is through its metabolites. Short-chain fatty acids (SCFAs) are well-known bacterial products derived from the gut microbiome (111). Butyrate, an important SCFA in the allo-HSCT context, produced by strain *Blautia*, was associated with better long-term outcomes for Gut-GVHD (112–114). In correlation with that, an increase in aGVHD severity was associated with a decrease in butyrate production (113, 115, 116). Moreover, lower circulating concentrations of SCFAs propionate and butyrate were found in day 100 plasma samples from cGVHD patients when compared with those who remained GVHD free (117). Mechanistically, GVHD improvement *via* butyrate in a mouse model is dependent upon the presence of SCFA receptor GRP43 (118). Additionally, butyrate ameliorated the metabolic defect of reduced succinate dehydrogenase A (SDHA) in allogeneic intestinal epithelial cells (IECs), which in turn reduced the severity of GVHD (119). Another study showed that high butyrate-producing organisms Clostridia increased intestinal Tregs, and those Tregs are involved in modulating gut inflammation response through several mechanisms (i.e., IL-10 release) (120, 121) that decreased Gut-GVHD and increased survival after allo-HSCT (113). Conversely, one study showed that the presence of butyrogenic bacteria after the onset of aGVHD was associated with subsequent SR-GVHD or cGVHD due to the ability of butyrate to inhibit human colonic stem cells from forming an intact epithelial monolayer (122). Therefore, butyrate may be beneficial or harmful in the pathogenesis of GVHD after the onset of aGVHD, depending on the condition of the mucosa.

In addition to the fiber-derived metabolites, SCFA, another metabolite derived from the intestinal microbiome known as indoles, an amino acid-derived metabolites, was found to reduce Gut-GVHD but did not have any impact on graft-versus-leukemia (GVL) activity in an experimental mouse model (123). Administration of indole-3-carboxaldehyde (ICA), an indole derivative, increased gene expression associated with type I interferon response, which has been shown to provide protection against radiation-induced intestinal damage and minimized GVHD pathology (123). Furthermore, the protective effect exerted by indoles seems to be mediated by Th17 responses in the intestinal tract and by IL-22-mediated effects on stem cells (124). Similar results were found in a human study: in a cohort of 131 adult patients receiving allo-HSCT, the study found that low levels of 3-indoxyl sulfate (3-IS) were associated with higher transplant-related mortality and worse outcome, mainly due to Gut-GVHD. The study also found that 3-IS urinary levels were correlated with gut microbiome diversity with the great presence of *Eubacterium rectale* and Ruminococcaceae, both taxa belonging to the Clostridia class (125). Another amino acid-derived metabolite, TMAO, has been identified as a fore for GVHD. TMAO augments alloreactive T-cell proliferation and Th1 subset differentiation mediated by the polarized M1 macrophages, resulting in higher severity of GVHD (126).

Apart from those well-studied microbiome compounds, researchers have indicated that polyamines, polycationic molecules produced by commensal bacteria, are important not only for Th subset fidelity (94) but also for epithelial proliferation and CX3CR1^+^ macrophage differentiation in the colon (127). Notably, in contrast to the decrease of SCFA and 3-IS after allo-HSCT, an increase of polyamine metabolites was found in recipients without GVHD (128, 129). Among the polyamine group, a significant increase of 5-methylthioadenosine (MTA) and *N*-acetylputrescine levels have been observed (128), which might promote gut integrity (130) *via* the inhibition of macrophage activation (131) and reduction of T-cell activation (132, 133), suggesting that polyamine metabolites might play a protective role in gut integrity in patients without Gut-GVHD. Of note, GCs were reported to inhibit the activity of ornithine decarboxylase (ODC), the rate-limiting enzyme in polyamine biosynthesis in acute lymphocytic leukemia cells (134, 135). However, the effects of polyamines in SR-Gut-aGVHD remain unclear. Recently, our study found that IL-22-dependent dysbiosis and reduction of CX3CR1^hi^ MNPs contributed to the pathogenesis of the SR-Gut-aGVHD in mice (46). It would be of interest to test whether GC treatment inhibits ODC activity and reduces polyamine metabolites in donor T cells as well as test whether supplementation of polyamines such as spermidine will benefit SR-Gut-aGVHD. It was also reported that the anti-inflammatory effect of GCs is Treg-dependent (136), and oral supplementation of a polyamine metabolite spermidine promoted Treg expansion (137).

In addition, a recent study has drawn increasing attention to the role of obesity in microbiota modulation during the development of GVHD. It has been demonstrated that obesity worsens experimental aGVHD associated with increased gut permeability, endotoxin translocation into the bloodstream, proinflammatory cytokine production, and reduced gut microbiota diversity. Treatment with antibiotics partially protected diet-induced obese (DIO) mice from lethal GVHD (138), and microbiome abnormalities caused by obesity appear to fuel the ongoing development of aGVHD (139). Consistent with previous observations, patients with a high body mass index had a decreased diversity of the gut microbiome and poorer overall survival following allo-HSCT (138). Taking these findings together, it is apparent that metabolically unhealthy body conditions related to microbiomes are important contributors to poor overall survival and GVHD outcomes. It would be of interest to investigate whether obesity is associated with SR-Gut-aGVHD in the future.

Considering the positive effects of the gut microbiome community and its metabolites on Gut-GVHD, fecal microbiota transplants (FMTs) have been evaluated as a therapy in patients suffering from SR-GVHD (140, 141). No adverse side effects have been reported, and most of the patients experienced various ranges of clinical benefits (114, 140, 142, 143). Nevertheless, all the reported studies involved only a small number of individuals. Additional studies with a large cohort should be designed to further investigate the effect of FMT on clinical outcomes for treating SR-Gut-aGVHD.

## Myeloid-Derived Suppressor Cells of Interest as a Therapeutic Option for Steroid Refractory-Gut-Acute Graft-Versus-Host Disease Patients

The MDSCs are an immunosuppressive population of myeloid cells that undergo systemic expansion in response to inflammation or cancer (144). In humans, MDSC can be subdivided into 3 subsets based on their characteristics: granulocytic polymorphonuclear leukocyte (PMN)-MDSCs defined by CD11b^+^CD14^−^CD15^+^, myeloid (M)-MDSC defined by CD11b^+^CD14^+^HLA-DR^−/lo^CD15^−^, and immature/early MDSC defined by CD33^+^ in the absence of lymphoid lineage and HLA-DR antigens. Generally, MDSCs are predominantly derived from either mouse BM or human PBMCs (145, 146).

During allo-HSCT, MDSCs have primarily been characterized by their ability to inhibit the proliferation of allogeneic T cells. Currently, in the murine GVHD model, four main mechanisms responsible for these immunosuppressive properties have been identified *in vitro* and *in vivo*: nitric oxide (NO) production, arginase 1-mediated l-arginine depletion, indoleamine 2,3-dioxygenase (IDO)-mediated tryptophan conversion, and promotion of T regulatory lymphocyte (i.e., Treg) survival (147–152). In human GVHD, some MDSC subsets were found to be associated with a lower incidence of aGVHD following allo-HSCT (153, 154). MDSCs isolated from patients early after allo-HSCT have been reported to suppress third-party CD4^+^ T-cell proliferation and Th1 differentiation while promoting Treg development (155).

Even though MDSCs effectively suppress alloimmune responses *in vitro*, their efficacy is limited in the context of aGVHD due to inflammasome activation. It was reported that during exposure to conditioning regimen and GVHD inflammatory environment, MDSCs lose their suppressor function in inhibiting GVHD lethality, which results from their induced conversion to a mature inflammasome-active state (156). Further study demonstrated that conditioning regimen-induced adenosine triphosphate (ATP) release plays a key role in MDSC dysfunction through the engagement of ATP receptors (P2x7R) and inflammasome activation of NLR family pyrin domain three (NLRP3); and inhibiting NLRP3 inflammasome activation and IL-1β secretion resulted in GVHD amelioration (157).

In terms of SR-Gut-aGVHD, the role of MDSCs has been poorly explored in either animal models or human studies. We found recently in our mouse model that the development of SR-Gut-aGVHD was associated with the reduction of donor-derived CD11b^+^CX3CR1^hi^ MNPs. Infusion of this population resulted in amelioration of Gut-aGVHD mediated by IFN-γ^−^IL-22^+^ alloreactive T cells (46), suggesting that donor-derived CD11b^+^CX3CR1^hi^ MNPs is likely to serve as MDSCs. It has been reported that M2 macrophages were enhanced in human SR-GVHD gut tissue when compared with both aGVHD at onset and normal biospy (47). Further study is required to determine whether the loss of CD11b^+^CX3CR1^high^ MNPs in our SR-Gut-aGVHD mouse model is associated with the expansion of M2 macrophages. We are further investigating the phenotype and function of the donor-derived CD11b^+^CX3CR1^hi^ MNPs in the mouse model of SR-Gut-aGVHD as well as in patients.

## Imaging Mass Cytometry Will Facilitate Progress in Steroid Refractory-Gut-Acute Graft-Versus-Host Disease Translational Research

Increasing insights into human immune responses that drive GVHD are closely connected with the technological advances that enable us to investigate immune response mechanisms at different levels including gene expression (e.g., single-cell RNA sequencing), epigenetic modification (e.g., ATAC sequencing), proteins (e.g., proteomics), tissue transcriptomics, dual host/microbe RNA-Seq, and intestinal organoid-based platform and cells (e.g., mass cytometry). Various tools have been utilized in a variety of human study areas including viral infection, autoimmune disease, and cancer (158–160). In addition, multiplexed imaging methods are becoming increasingly important not only for basic science research but also for clinical research (161–166).

Tissue transcriptomics was proven invaluable for describing heterogeneous cell populations within GVHD target organs in both non-human primate (NHP) aGVHD model and human GVHD studies (47, 167). Dual RNA-sequencing that utilizes RNA-Seq applications enabled by next-generation sequencing (NGS) can be used to investigate transcriptional changes in both infected bacteria and host cells. Dual RNA-Seq can provide unique insights into bacterial infection processes and corresponding host responses by simultaneously examining both organisms from an individual biological sample (168–170). Both tissue transcriptomics and dual host/microbe RNA-Seq have been applied to human SR-GVHD study and led to the discovery of T cell-independent mechanisms in mediating SR-GVHD (47). Meanwhile, it has been found that donor T cells or TNF-α induced death of cultured human organoids was achieved by utilizing an *ex vivo* intestinal organoid-based platform (171). Therefore, the *ex vivo* organoid culture systems can serve as a platform for testing pathogenic or protective lymphocytes that are involved in SR-GVHD development or in selecting drug candidates for prospective therapy (172).

On the other hand, imaging mass cytometry (IMC) is another new technique that not only allows for tissue transcriptomics analysis but also can help us locate and visualize the complex immune mediators in a GVHD target organ such as the intestine without disturbing the original tissue structure, and it allows imaging of the immune system at subcellular resolution (173). With IMC, it is now possible to examine 30–40 parameters on a single tissue section at one time, and this capability is extremely relevant for several applications including cancer and diabetes research, as well as the determination of complex immune subsets in a specific tissue microenvironment (174–178). The development of IMC represents a landmark technological advance since it allows for increased numbers of markers to be stained, and they can be acquired and visualized simultaneously as compared to traditional tissue imaging technology. More importantly, the IMC platform enables the analysis of numerous cell types in their native microenvironment concurrently, which consists of a complex matrix of fluids, proteins, and cells. The evaluation of a certain cell in its respected microenvironment determines how it is phenotypically characterized and how it functions within a given organ in both healthy and pathological states (179, 180).

To this point, the primary application of IMC has been in the study of cancer and autoimmune diseases such as type 1 diabetes (174–176). IMC utilization in those areas provided helpful information regarding the location and interactions between multiple subsets of cells. Up to now, some GVHD mechanism studies with patient biopsy tissues suffer from limited tissue sample size and limited markers (only 2–5) that traditional immunochemistry or immunofluorescent staining can achieve. Therefore, the application of IMC will likely become an indispensable tool for atlas studies of immune cell composition, interactions, and anatomical location *in situ*, which will further characterize the immune networks that are active during various stages of GVHD including SR-Gut-aGVHD. Nevertheless, tread lightly when using this technology especially when converting raw IMC data to the high-dimensional plot by cell segmentation, particularly in defining “novel” cell clusters. Since IMC offers a large number of parameters, the prospect of identifying potential “novel” cell subsets is indeed very tempting. The underlying problem of this cell segmentation feature remains imperfect because it is often affected by the different target antigen signal performances in the distinct tissue microenvironment, potentially leading to incorrect classification of new cell phenotypes. At best, the phenotypic information provided by IMC should be further validated using more robust single-cell techniques such as flow or mass cytometry (181). Through merging IMC with other high-dimensional assays, we anticipate that IMC will be vital to our understanding of the etiology of SR-Gut-aGVHD as well as to our ability to make rapid clinical decisions.

## Conclusions

**Figure 1:**
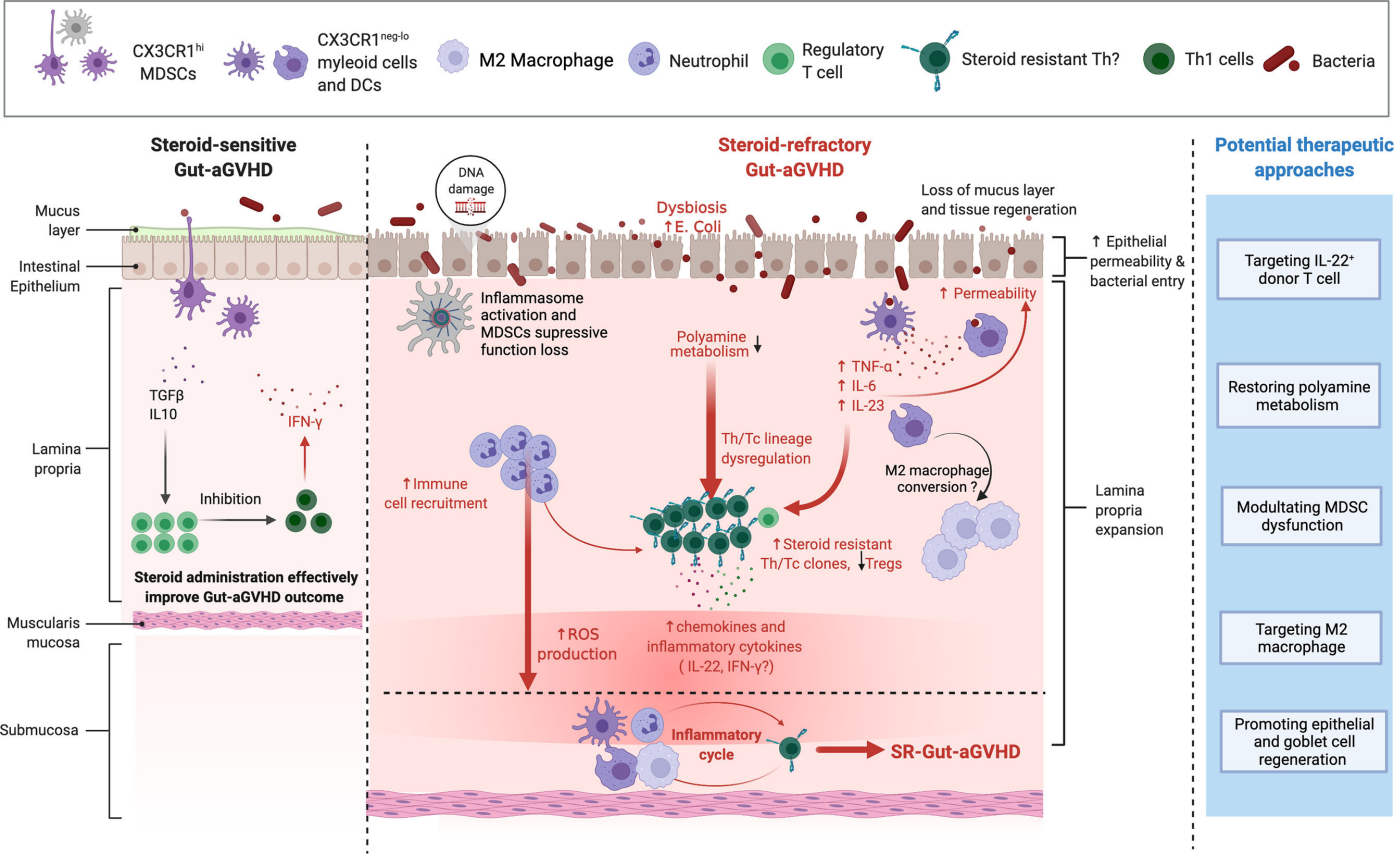
Mechanism of steroid-refractory acute gut graft-versus-host disease (GVHD) (SR-Gut-aGVHD) and potential therapeutic approaches. With tissue damage due to conditioning, prolonged glucocorticoid (GC) treatment causes dysbiosis with expansion of pathogenic bacteria *Escherichia coli*. GC treatment may inhibit ornithine decarboxylase (ODC) activity in donor T cells resulting in the reduction of polyamine metabolites. At the same time, dysbiosis alters the microbiome metabolites, which may also lead to the reduction of polyamines. The reduction of polyamine metabolites causes Th lineage dysregulation and expansion of steroid-resistant Th/Tc clones that produce multiple proinflammatory cytokines (e.g., IL-22 and IFN-γ), along with the reduction of Tregs. Proinflammatory cytokines and chemokines such as IL-22 and IFN-γ attract neutrophils and myeloid cells to infiltrate colon tissues. The presence of inflammatory cytokines, tissue DNA damage, and the activation of inflammasomes cause CX3CR1^hi^ mononuclear phagocytes (MNPs) to become inflammatory CX3CR1^lo/−^ cells and subsequently drive myeloid-derived suppressor cells (MDSCs) to lose their ability to suppress inflammation. Loss of protective mucus layers, increase of intestinal epithelial permeability, and dysfunctional CX3CR1^hi^ MNPs allow bacterial invasion into the tissues, further triggering the production of inflammatory cytokines such as TNFα, IL-6, and IL-23, which further expand the steroid-resistant Th clones. In addition, GC treatment can also augment neutrophil migration and infiltration into the intestine and cause tissue damage *via* reactive oxygen species (ROS) production. Therefore, under prolonged GC treatment, the infiltrating T cells that produce IL-22 and/or IFN-γ, as well as proinflammatory dendritic cells (DCs) and CX3CR1^neg-/lo^ myeloid cells and neutrophils, form a feed-forward pathogenic inflammatory cycle, resulting in full-blown SR-Gut-aGVHD. Based on these mechanisms, we propose the following potential therapeutic approaches: 1) targeting IL-22-producing donor T cell; 2) restoring polyamine metabolism; 3) modulating MDSC dysfunction; 4) targeting M2 macrophage; and 5) promoting epithelial and goblet cell regeneration. Figure is created with BioRender.com.

In conclusion, according to the findings from basic immunology and the experimental murine model of SR-Gut-aGVHD, we would like to highlight the following points: a) cytokines produced by specific Th subsets have distinct sensitivity to GC treatment in the setting of SR-Gut-aGVHD, and the GC-insensitive cytokines such as IL-22 contribute to the pathogenesis of the disease. b) During the development of SR-Gut-aGVHD, the evolution of steroid-resistant T-cell clones is likely to be characterized as co-expression of multiple inflammatory cytokines. c) IL-22 serves a dual role of being proinflammatory and anti-inflammatory, respectively, depending on the context of the immune microenvironment. d) Dysbiosis contributes to the pathogenesis of SR-Gut-aGVHD, and further investigation into the role of microbiome metabolites will improve our understanding of the interaction between the microbiome and immune response in the setting of SR-Gut-aGVHD. e) MDSCs potentially could regulate alloreactive T-cell response in the context of SR-Gut-aGVHD. In this review, we would like to propose the prospective mechanisms and potential therapeutic approaches for SR-Gut-aGVHD pathogenesis, as depicted in **Figure 1**. Additional studies are needed to further define the transcriptional networks and epigenetic programs that reinforce the functional state of T cells’ resistance to GCs. In addition, incorporating advanced technology like IMC to assess the immune cross talk between T cells, MDSCs, and microbiome metabolites will help us identify potential therapeutic interventions.

## Author Contributions

QS wrote the review manuscript. UN edited the manuscript. DZ critically reviewed and edited the manuscript. All authors listed have made a substantial, direct, and intellectual contribution to the work and approved it for publication.

## Funding

This work was supported by the National Institutes of Health Grant R01 CA228465 (to DZ) and National Natural Science Foundation of China grant no. 82100226 (to QS).

## Conflict of Interest

The authors declare that the research was conducted in the absence of any commercial or financial relationships that could be construed as a potential conflict of interest.

## Publisher’s Note

All claims expressed in this article are solely those of the authors and do not necessarily represent those of their affiliated organizations, or those of the publisher, the editors and the reviewers. Any product that may be evaluated in this article, or claim that may be made by its manufacturer, is not guaranteed or endorsed by the publisher.
